# Bacterial Delivery of Nuclear Proteins into Pluripotent and Differentiated Cells

**DOI:** 10.1371/journal.pone.0016465

**Published:** 2011-01-27

**Authors:** Candace Bichsel, Dennis K. Neeld, Takashi Hamazaki, Donghai Wu, Lung-Ji Chang, Lijun Yang, Naohiro Terada, Shouguang Jin

**Affiliations:** 1 Department of Molecular Genetics and Microbiology, College of Medicine, University of Florida, Gainesville, Florida, United States of America; 2 Department of Pathology, College of Medicine, University of Florida, Gainesville, Florida, United States of America; 3 Key Laboratory of Regenerative Biology, Guangzhou Institute of Biomedicine and Health, Chinese Academy of Sciences, Guangzhou, China; University of California Merced, United States of America

## Abstract

Numerous Gram negative pathogens possess a type III secretion system (T3SS) which allows them to inject virulent proteins directly into the eukaryotic cell cytoplasm. Injection of these proteins is dependent on a variable secretion signal sequence. In this study, we utilized the N-terminal secretion signal sequence of *Pseudomonas aeruginosa* exotoxin ExoS to translocate Cre recombinase containing a nuclear localization sequence (Cre-NLS). Transient exposure of human sarcoma cell line, containing Cre-dependent *lacZ* reporter, resulted in efficient recombination in the host chromosome, indicating that the bacterially delivered protein was not only efficiently localized to the nucleus but also retained its biological function. Using this system, we also illustrate the ability of *P. aeruginosa* to infect mouse embryonic stem cells (mESC) and the susceptibility of these cells to bacterially delivered Cre-NLS. A single two-hour infection caused as high as 30% of the mESC reporter cells to undergo loxP mediated chromosomal DNA recombination. A simple antibiotic treatment completely eliminated the bacterial cells following the delivery, while the use of an engineered mutant strain greatly reduced cytotoxicity. Utility of the system was demonstrated by delivery of the Cre-NLS to induced pluripotent stem cells to excise the floxed oncogenic nuclear reprogramming cassette. These results validate the use of T3SS for the delivery of transcription factors for the purpose of cellular reprogramming.

## Introduction

Bacteria possess an arsenal of virulence factors used to moderate eukaryotic cells. One such mechanism utilized by many Gram negative bacteria is the type III secretion system (T3SS). This transmembrane needle-like projection from the bacterial membrane allows these pathogens to inject proteins across the eukaryotic cell membrane, bypassing endocytic pathways [1], [2]. While the type III secretion system itself is relatively conserved among bacterial species, the secreted effectors have diverse biological functions and typically modulate key host regulatory proteins to promote bacterial infection [3]. The cytotoxic proteins delivered by this system are guided to the injectisome by a variable N-terminal signal sequence [4]–[7].

*Pseudomonas aeruginosa* is a ubiquitous opportunistic pathogen, which secretes relatively few exotoxins by a single T3SS [3]. Type III secretion is highly regulated in *P. aeruginosa*, and can be induced *in vitro* by low extracellular calcium levels or direct host cell contact [8], [9]. Once activated, *P. aeruginosa* secretes three of four exotoxins: ExoS and ExoT, which possess both ADP ribosyltransferase and GTPase activating protein activity [10]; ExoY, an adenylyl cyclase [11]; and ExoU, a lipase with hemolyic activity [12], [13]. Ultimately, injection of these toxins results in host cell rounding and death, rendering the bacterial survival advantage within the host environment. Of these effectors, the functional domains of ExoS are best characterized. Previous studies have shown that various lengths of the N-terminus of ExoS can be fused to exogenous proteins and direct them for injection into the host cell cytosol in a type III secretion dependent manner [5], [6]. While one such study has demonstrated the functionality of these injected fusion proteins by *ex vivo* complementation of a cytoplasmic protein deficiency [6], the T3SS has not yet been applied to the delivery of nuclear proteins.

The development of a simple, efficient system for introduction of nuclear proteins would meet an emerging need which has been made quite apparent in recent studies. The ability to reprogram terminally differentiated nuclei to a pluripotent state by forced expression of key transcription factors (Oct4, Sox2, cMyc, Klf4) has been a remarkable breakthrough in molecular and cell biology [14]–[16]. However, the therapeutic application of these reprogrammed cells (iPS cells) is severely hindered by the integration of oncogenic transgenes. There have been numerous attempts to overcome this limitation, including the use of DNA reprogramming cassettes which can be excised by Cre recombinase once cells have been stably reprogrammed [17], [18]. Cre is a site specific, bacteriophage derived recombinase which begets homologous recombination between sequences known as LoxP sites [19]. A DNA sequence flanked by direct repeat of LoxP sites will be excised upon Cre mediated recombination. This Cre-loxP system is widely used in modern molecular biology and is particularly useful in the generation of conditional gene knockouts [20].

In this report, we describe the use of the *P. aeruginosa* T3SS as an alternative method to deliver functional Cre recombinase to the nuclei of differentiated and pluripotent cells, achieving DNA recombination through loxP sites on the chromosome, resulting in alteration of host cell gene expression. Neither the transient bacterial infection nor the bacterially delivered Cre affected the pluripotency of the mouse ES cell or iPS cells. This study paves the way for future application of this novel protein delivery technology in therapeutic cellular reprogramming, as this is a safe alternative to the current gene delivery mediated reprogramming method.

## Results

### Generation of a *P. aeruginosa* strain for protein delivery

**Figure 1:**
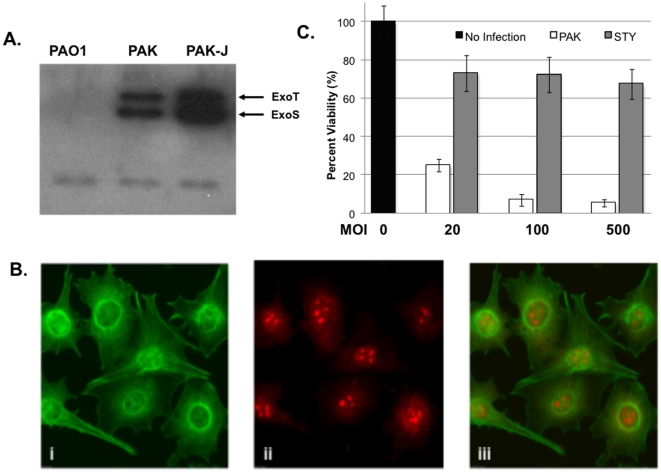
Protein delivery by type III secretion system of *P. aeruginosa*. (**A**) Comparison of ExoS secretion by standard laboratory strains of PAO1, PAK and a hyper secreting strain PAK-J. Strains were grown under type III secretion inducing conditions and culture supernatants were subjected to Western blot analysis using anti-ExoS antibody which recognizes both ExoS and ExoT. (**B**) Immunohistochemistry of MEF cells following infection by PAK-J (60 min at MOI 20). Cells were stained with anti-ExoS antibody followed by FITC labeled secondary antibody; (i) visualization of ExoS; (ii) visualization of nuclei with Propidium Iodine stain; (iii) compilation of (i) & (ii). (**C**) Comparison of MEF viability after 3 hour infection with PAK-J or PAK-JΔ*STY* at indicated MOIs.

The standard laboratory strain of *P. aeruginosa* (PAO1), whose genome has been sequenced, secretes low levels of type III effectors under type III inducing conditions. To identify a strain with elevated type III secretion, we screened *P. aeruginosa* strains in our collection, including commonly used laboratory strains as well as clinical and environmental isolates. Interestingly, a laboratory strain of PAK that had been passaged in our laboratory for over 10 years displayed the highest level of ExoS secretion under type III inducing conditions (hereon referred to PAK-J) (Fig. 1A). According to quantitative ELISA assays [9], this strain secretes more than 10 times higher level of ExoS than the standard laboratory strain PAK. As expected, infection of cultured mammalian cells by this strain caused significantly higher cytotoxicity, resulting in complete rounding and lifting of varius adhering cells, such as HeLa and mouse embryonic fibroblast (MEF), within 3 hours at an MOI of 20. Accounting for this, T3SS injected bacterial effector protein ExoS can be detected in 100% MEF cells under a similar infection condition, with injected ExoS mainly localized around paranuclear region (Fig. 1B). The genetic alterations in PAK-J that are responsible for elevated type III secretion are actively being investigated.

The *P. aeruginosa* strain PAK-J expresses three type III secreted effectors, ExoS, ExoT and ExoY, potent exotoxins that account for much of the cytotoxicity associated with this bacterium. In order to maximize protein injection, the delivery strain must be capable of prolonged incubation with host cells. As such, each of the three genes was deleted from the PAK-J chromosome by successive unmarked allelic exchange. The resulting strain, PAK-J*ΔSTY*, has comparatively reduced cytotoxicity, as assessed by the number of adhered HeLa cells following the bacterial infections (Fig. 1C). The assay results indicated that deletion of the PAK-J type III secreted exotoxins reduces cytotoxicity by approximately 70%, which is consistent with the assay result of lactate dehydrogenase release assay (data not shown). This diminution in cytotoxicity will allow for increased infection duration, and thus enhanced protein delivery by the type III secretion.

Following protein delivery, it is essential to completely eliminate the residual bacterial cells to avoid contamination. Various combinations of antibiotics were tested, including penicillin, streptomycin, spectinomycin, gentamicin and ciprofloxacin. The most effective combination was gentamicin with ciprofloxacin. In a 6-well plate, HeLa cells were infected with PAK-J*ΔSTY* at MOI 100 for 3 hours, freely floating bacterial cells were removed by repeated washes with PBS, then the HeLa cells were grown in DMEM+5% FBS supplemented with 200 µl/ml gentamicin and 20 µl/ml cirofloxacin. After 3 hours, majority bacterial cells were killed with occasional recovery of 1–10 bacterial cells while no viable bacterial cells were detected by 24 hours, as determined by plating the whole HeLa cell lysates on L-agar plates. Further confirming this, after the initial 24 hours of treatment with antibiotics, the HeLa cells could be cultured in an antibiotic-free medium for three days without bacterial contamination, demonstrating effective elimination of the bacterial delivery cells. Most importantly, neither the HeLa cell morphology nor its growth rate has changed after subjecting to the bacterial infection and clearance by antibiotics.

### Injection of Cre recombinase through the bacterial T3SS

The bacteriophage derived Cre recombinase is a widely used genetic tool which allows for excision of DNA between two LoxP sequences by homologous recombination [19]. The Cre-Lox system was chosen to demonstrate the utility of this bacterial protein delivery system primarily because it is a DNA-interacting protein which must localize to the nucleus to exert its recombinase activity. Additionally, there are many Cre-dependent reporter cell lines readily available, making this a very convenient assay system.

**Figure 2:**
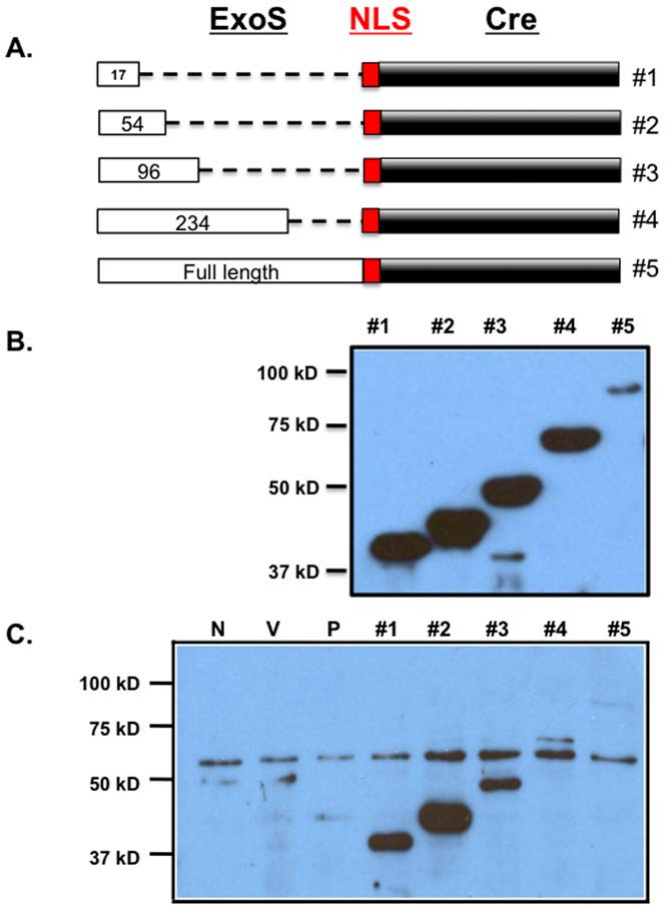
Injection of indicated ExoS-Cre fusions. (**A**) Cre recombinase was fused in frame with various N-terminal portions of ExoS, with a nuclear localization sequence (NLS) in the fusion junction. (**B**) Detection of the five ExS54-Cre fusion proteins in PAK- JΔ*STY* harboring the corresponding fusion constructs. Bacterial cells were grown under type III inducing condition and cell lysates were subjected to Western blot using anti-Cre antibody. (**C**) TE26 cells were infected with PAK-JΔ*STY* containing the aforementioned constructs, lysed, and subject to Western Blot with anti-Cre antibody. N, no infection control; V, vector control, PAK-JΔ*STY*/pUCP20; P, T3SS mutant control, PAK-JΔ*popD*/pExoS54-Cre; Lanes1-5 are Cre fusions to ExoS17, ExoS54, ExoS96, ExoS234 and ExoS full length, respectively.

To determine the optimal signal sequence for delivery of the Cre, we generated fusions of various lengths of ExoS to Cre recombinase, with the addition of a nuclear localization sequence in the fusion junction (Fig. 2A). Each construct was then introduced into PAK-J*ΔSTY* and tested for proper expression under type III inducing condition as well as efficiency of T3SS mediated injection into host cells. PAK-J*ΔSTY* strains containing the various fusion constructs were grown in L-broth plus 5 mM EGTA for 3 hours to induce type III secretion system genes. Cell pellets were separated on SDS-PAGE and subjected to Western Blotting with an antibody against Cre recombinase. As indicated in Figure 2B, all fusion proteins were made equally, except for the full length ExoS fusion which was produced at low level. Next, a human sarcoma cell line (TE26) was infected with PAK-J*ΔSTY* containing the various length Cre recombinase fusions at an MOI of 50 for 2 hours. As the injection assay results show in Fig. 2C, while the first 17 amino acids of ExoS appear to be sufficient, 54 amino acids directed Cre recombinase for type III secretion more efficiently. Secretion was gradually reduced as the ExoS portion increased beyond 54 amino acids long. To verify that ExoS54-Cre injection occurs in a type III specific manner, we also expressed this construct in a type III defective strain, PAK- J*ΔpopD* [21], and saw no intracellular Cre recombinase upon TE26 infection (Fig. 2C).

### Functional analysis of bacterially delivered Cre protein

**Figure 3:**
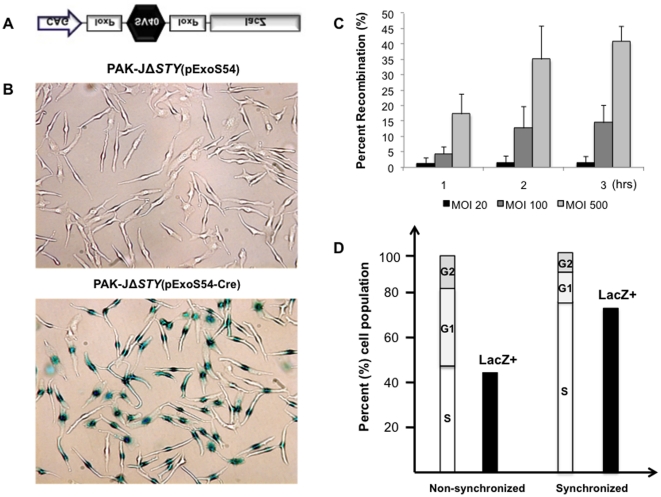
Assessment of Cre recombinase activity. Cre function was assessed by LacZ positive TE26 cells which contain a floxed terminator preventing *lacZ* expression (**A**). TE26 cells were infected for various times and MOIs and subsequently stained with X-gal for β-galactosidase (**B**) to determine the optimal infection conditions as indicated by percentage LacZ positive cells (**C**). TE26 cells were infected at an MOI of 50 for 3 hours before or after cell cycle synchronization and X-gal stained for β-galactosidase activity (**D**).

To assess the functionality of the fusion protein, we have employed the TE26 cell line, which contains a floxed SV40 transcriptional terminator that prevents downstream *lacZ* expression (Fig. 3A). Upon Cre mediated recombination, the DNA between loxP sites is removed, allowing *lacZ* expression which can be evaluated by β-galactosidase activity.

TE26 cells were infected with PAK-JΔSTY(pExoS54-Cre) at various MOIs (20, 100, 500) for 1–3 hours. The infections were cleared and cells were allowed 48 hours to undergo recombination and *lacZ* expression. Cells were then fixed and stained with a solution containing bromo-chloro-indolyl-galactopyranoside (X-gal) to assess β-galactosidase activity. As the *lacZ* reporter gene contains a nuclear localization sequence, the β-galactosidase activity was mainly observed inside the TE26 nuclei (Fig. 3B). The percentage of β-galactosidase positive cells increases in a dose and time dependent manner, with infection at an MOI of 500 for 3 hours resulting in the highest (42%) efficiency (Fig. 3C). The ability of bacterially delivered ExoS54-Cre to induce such levels of recombination indicates that the process of type III secretion and the presence of the ExoS54 signal sequence interfere with neither the nuclear localization nor the biological function of Cre recombinase.

Based on imunocytochemical staining of the translocated proteins, nearly 100% of cells are normally injected by the bacterially delivered proteins (Fig. 1B), however, the recombination efficiency is much lower than 100%. It is conceivable that Cre-mediated DNA recombination can only occur when the target loxP sites are freely accessible, most likely during the DNA replication in S phase. Indeed, the maximal 42% LacZ positive cells observed following infection of the TE26 cells correlated with 47% of S phase cells in the cell population, where G1/G0 and G2/M phase cells were 34% and 19%, respectively, as determined by FACS analysis of PI stained cells (Fig. 3D). To test this further, TE26 cells were synchronized by a double thymidine blocking method, obtaining cell population with 78% of the TE26 cells in S phase while G1/G0 and G2/M phase cells were 17% and 5%, respectively. Infection of the synchronized cells with the PAK-J*ΔSTY*(pExoS-Cre) for 3 hours at an MOI 500 resulted in 75% LacZ positive cells (Fig. 3D). Clearly, the proportion of *lacZ* positive cells increases with the increased number of cells in S phase, thus the Cre mediated recombination is significantly influenced by the chromosome structure.

### Delivery of functional nuclear proteins to mESC

**Figure 4:**
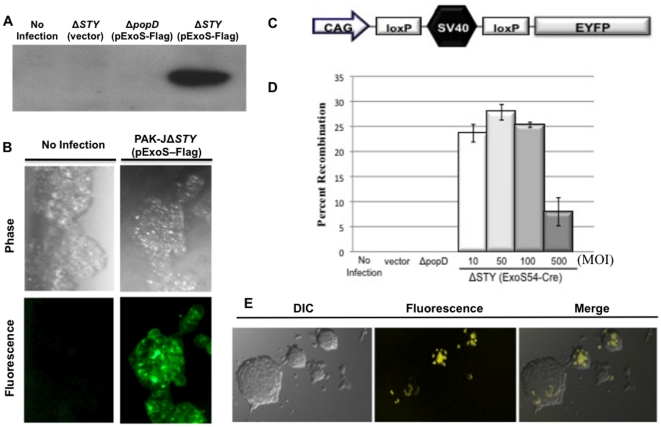
Protein injection into mouse embryonic stem cells (mESC). (**A**) mESC were infected with PAK-J strains at a MOI of 50 for 2.5 hours, lysed and assayed for injected ExoS-Flag by anti-Flag Western Blot. (**B**) mESC were infected with PAK-JΔ*STY*(pExoS-Flag) for 2.5 hours and subsequently fixed and immunostained with anti-Flag to illuminate translocated ExoS-Flag protein. (**C**) R26R-EYFP mESC cells contain a floxed terminator preventing downstream EYFP expression. (**D**) R26R-EYFP were infected with PAK-JΔ*STY*(pExoS54-Cre) at various MOIs for 2.5 hours. Cells were collected for FACS analysis 48 hours post infection. (**E**) EYFP-positive mESC were plated after infection to assess EYFP expression by confocal fluorescence microscopy.

Given the rise in the use of stem cells in many fields of biological and pharmaceutical research, a proficient protein delivery system should be applicable to both differentiated and pluripotent cells. While the ability of *P. aeruginosa* to infect an assortment of cell types *in vitro* has been well established, the susceptibility of pluripotent stem cells to *P. aeruginosa* infection remains undetermined. In confirmation, mESC were infected with PAK-J*ΔSTY*(pExoS-Flag), which expresses a catalytically inactive, Flag-tagged version of ExoS. Subsequent Western blot of the cell lysate illustrated clearly translocated ExoS-Flag (Fig. 4A). The translocation occurred in a type III secretion dependent manner as PAK-J*ΔpopD*(pExoS-Flag), which is defective in type III secretion, was unable to deliver the ExoS-Flag. These results are further substantiated by immunocytochemical staining of PAK-J*ΔSTY*(pExoS-Flag) infected mESC with an anti-Flag antibody (Fig. 4B). Together these data suggest that mESC are also receptive to *P. aeruginosa* mediated protein delivery.

Considering the notorious difficulty of genetic manipulation in stem cells, it is necessary to determine the ability of type III secreted proteins to localize to the mESC nucleus and maintain function. To quantitatively evaluate the functionality and efficiency of protein delivery to mESC, we utilize a reporter cell line, R26R-EYFP mESC, which contains a floxed SV40 terminator preventing downstream expression of Enhanced Yellow Fluorescent Protein (EYFP) in the Rosa26 locus (Fig. 4C) [22]. Similar to the TE26 reporter, Cre mediated recombination will allow for EYFP expression which can be visualized by fluorescence microscopy and quantified by flow cytometric analysis. R26R-EYFP mESC were co-incubated with PAK-J*ΔSTY*(pExoS54-Cre) at various MOIs for 2.5 hours, as this duration had been determined optimal in preliminary trials (data not shown). Cells were subjected to FACS analysis 48 hours post-infection. The efficacy of ExoS54-Cre mediated recombination is represented as the percentage of EYFP positive cells in the infected mESC population (Fig. 4D). Similar to the TE26 results, PAK-J*ΔSTY*(pExoS54-Cre) induced recombination occurs in a type III secretion specific, dose dependent manner (Fig. 4E). Recombination efficiency peaks with an MOI of 50, resulting in nearly 30% EYFP positive cells, and steadily declines at higher MOIs, possibly due to increased cytotoxicity. After sorting, EYFP positive cells were plated on gelatin coated glass coverslips for observation of EYFP expression as well as cellular morphology (Fig. 4F). The ability of these previously infected cells to form colonies characteristic of mESCs and maintain similar growth rate suggests that neither bacterial infection nor bacterial protein mediated alteration in gene expression have significant effects on the pluripotency of these exceptionally sensitive cells.

### Excision of reprogramming gene cassette from iPSC by bacterially injected Cre protein

**Figure 5:**
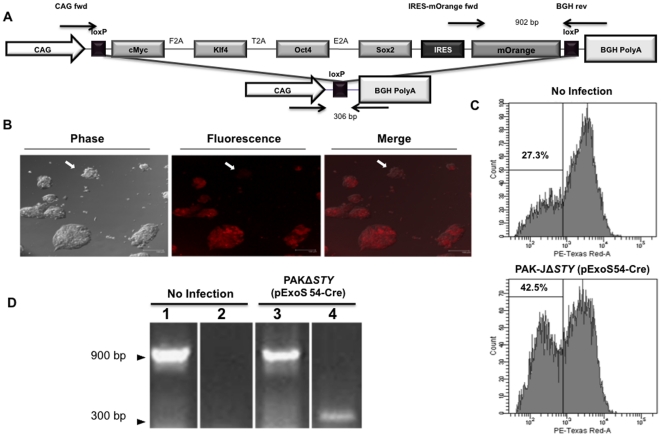
ExoS54-Cre mediated excision of an iPSC nuclear reprogramming cassette. TNGim05 iPSC contain a floxed reprogramming cassette (**A**). ExoS54-Cre mediated recombination results in loss of mOrange expression, which can be observed by fluorescence confocal microscopy (**B**) and flow cytometry (**C**). Reprogramming cassette excision is confirmed by PCR analysis (**D**). Lanes 1 and 3, IRES primer set, lanes 2 and 4, CAG primer set.

Induced pluripotent stem cells (iPSC) have been generated by forced expression of four transcription factors Oct4, Sox2, c-Myc, and Klf4, which are upregulated in embryonic stem cells [14]. Exogenous expression of these factors activates stable expression of the endogenous pluripotency genes, relinquishing transgene dependency [17]. While nuclear reprogramming is a breakthrough in the field of stem cell biology, it is primarily criticized for the lingering oncogenic transgenes that remain integrated in the chromosome of iPSC. In order to circumvent this issue, a nuclear reprogramming cassette was developed to contain all four transcriptional factors and a mOrange fluorescent reporter flanked by loxP sites (Fig. 5A), such that the cassette can be removed by Cre mediated recombination following stable reprogramming. TNGim05 mouse embryonic fibroblast derived iPSC were generated by integration of the single reprogramming cassette, which has yet to be excised [17]. As such, these cells display mESC-like morphology and mOrange expression when cultured *in vitro*. Upon recombination, the reprogramming cassette will be excised, and the iPSC will maintain pluripotency, but lose mOrange expression.

The TNGim05 iPSC were infected at an MOI of 50 for 2.5 hours, as this condition maximized ExoS54-Cre mediated recombination in R26R-EYFP mESC (Fig. 4D). Cells were allowed 48 hours to undergo recombination prior to FACS analysis. Sorted cells were evaluated for recombination as indicated by percentage of decrease in mOrange positive cells (Fig. 5B). While both infected and uninfected TNGim05 iPSC samples contain an mOrange negative population, there is a significant increase with PAK-J*ΔSTY*(pExoS54-Cre) infection (Fig. 5C), indicating a subpopulation of the negative cells have actually undergone recombination. Cassette excision was verified by PCR analysis of pooled mOrange negative TNGim05 iPSC cells (Fig. 5D). In consistent with the fluorescence microscopic imaging, PCR results demonstrate that while not the entire mOrange negative population underwent recombination, there was a substantial fraction of cells having had the reprogramming cassette removed in an ExoS54-Cre dependent manner. Furthermore, the mOrange negative cells propagated normally in ES medium, forming colonies characteristics of iPS cells, suggesting transient exposure to the bacteria did not alter the iPS cell characters.

## Discussion

The use of the bacterial type III secretion system for transient delivery of nuclear proteins to modify intrinsic gene expression has the potential to replace many of the current methods that have several pitfalls such as inefficient, mutagenic and clinically inapplicable. To illustrate the utility of this system, we have generated a strain of *P. aeruginosa* with diminished cytotoxicity to deliver Cre, a well characterized protein which functions only in the nucleus to interact with DNA and exerts its recombinase activity. The availability of numerous Cre-dependent reporters has conveniently allowed us to observe the effects of Cre delivery to both differentiated and pluripotent cells. The ability of bacterially delivered ExoS54-Cre to induce high levels of β-galactosidase activity in TE26 cells indicates that the process of type III secretion and the presence of the ExoS54 signal sequence do not interfere with the ability of this protein to properly localize to the nucleus of host cells or exert its biological function.

Our experimental data support the notion that Cre-mediated DNA recombination can only occur when cells are in S phase or when the target loxP sites are freely available, as evident from the observation that the LacZ positive recombinant TE26 cells increase in proportion to the S phase cells during the Cre delivery by the bacteria. For TE26 cells, one full cell cycle takes around 34 hours to complete under our culture condition and a transition time from G2 to S phase takes about 20 hours, thus to achieve close to 100% efficiency of recombination, the injected Cre protein needs to be stable for a time period when all of the cells go through the S phase, which is at least 20 hours for TE26 cell. Apparently, the intracellular stability of the injected ExoS54-Cre is much shorter than the required 20 hours, presumably due to proteasome mediated breakdown. It is possible to achieve 100% Cre-mediated recombination by combining several approaches, including (i) an increase of S-phase cell fraction by synchronization, (ii) an increase of the half life of the translocated Cre by utilizing a proteosome inhibitor, and (iii) successive infections for Cre to encounter S phase for every cell in the population.

While embryonic stem cells are notoriously sensitive [23], the presence of EYFP positive colonies after bacterial delivery of ExoS54-Cre indicates that these cells are susceptible to *P. aeruginosa* infection, but neither infection nor protein delivery significantly affects the cellular morphology of R26R-EYFP mESCs. This finding is in accordance with the results of a recent study which demonstrates that mESC infection with other T3SS expressing bacteria, such as *Shigella flexneri,* does not alter pluripotency, as evaluated by Oct4 expression levels [23]. Given the inherent difficulty of manipulating embryonic stem cells, we have achieved relatively high delivery efficiency in a short time (2–3 hours) with a single infection. The infection efficiency may further be increased with multiple rounds of protein delivery. We are currently investigating the proficiency of repeated protein delivery to modify gene expression. In so doing, we are examining the long-term effects of bacterial infection on host cell characters.

We observed a dose-dependent increase in the efficiency of ExoS54-Cre-mediated recombination, which peaked around 30% at a MOI of 50 for R26R-EYFP mESC. The subsequent decline in recombination is clearly not due to insufficient protein delivery, but more likely resultant of excess protein translocation or bacterial cytotoxicity. Deletion of the *P. aeruginosa* type III secreted exotoxins resulted in a considerable decrease in cytotoxicity, as compared to that of wild-type PAK-J strain, which allowed cells to remain viable and undergo gene expression changes after infection. However, *P. aeruginosa* possesses additional virulence factors that contribute to cytotoxicity. Efforts are currently underway to further reduce the toxicity of this strain in an attempt to enhance cell viability and the efficacy of protein delivery. In addition to reduction of cytotoxicity, we are also in the process of engineering a strain which is more sensitive to antibiotics. Currently, gentamicin and ciprofloxacin are used to eradicate any residual bacteria after infection. While bacterial survival assays have indicated that these conditions are sufficient to destroy lingering intracellular and extracellular bacteria, it will be more convenient to infect with a strain that is sensitive to antibiotics commonly used in cell culture, such as penicillin and streptomycin. Alternatively, an auxotrophic mutant can also be utilized in which specific nutrient withdrawal will result in inhibition of the bacterial growth.

These studies serve as a foundation for the bacterial delivery of transcription factors to efficiently modulate concentration-dependent and temporal activation of gene expression to direct cell fate switch without jeopardizing genomic integrity which is critical for future clinical translation. The ability of few exogenous transcription factors to completely redirect endogenous gene expression is epitomized by the discovery of nuclear reprogramming. Induction of pluripotency, while almost exclusively achieved by transgene expression, has been documented to occur with recombinant protein transduction as well, albeit at extremely low efficiency [24], [25]. Having demonstrated the ability to deliver large quantities of nuclear targeting protein directly into eukaryotic cells, efforts are currently underway to harness the power of the type III secretion system to deliver nuclear reprogramming factors to efficiently induce pluripotency and lineage specific differentiation.

## Materials and Methods

### Bacterial strains and plasmids

The bacterial strains and plasmids used in this study are listed in Table 1. *P. aeruginosa* and *E. coli* were grown in Luria (L) broth or on L agar plates at 37°C. Antibiotics were used at a final concentration of 150 µg carbenicillin, or 100 µg tetracycline per ml for *P. aeruginosa* and 100 µg ampicillin per ml for *E. coli*.

**Table 1:** Strains and plasmids used in this study.

Strain or plasmid	Description	Source
*P. aeruginosa*		
PAO1	*P. aeruginosa* laboratory strain	[30]
PAK	*P. aeruginosa* laboratory strain	David Bradley
PAK-J	PAK derivative with enhanced T3SS	This study
PAK-JΔ*popD*	PAK with chromosomal deletion of the *popD* locus	This study
PAK-JΔ*STY*	PAK with chromosomal deletion of *exoS, exoT, exoY* loci	This study
*E. coli*		
S-17	Strain expressing DNA mobilization genes	[31]
DH5α	F^−^ φ80Δ*lac*ZΔM15 *end*A1 *rec*A1 *hsd*R17(m_k_-m_k_-) *sup*E44 *thi*-1 *rel*A1 Δ(*lac*ZYA-*arg*F) *gyr*A96 *deo*R	[32]
Plasmids		
pUCP20	*Escherichia-Pseudomonas* shuttle vector; Ap^r^ (Cb^r^)	[33]
pExoS17-Cre	17 aa of ExoS fused to nuclear Cre recombinase in pUCP20; Cb^r^	This Study
pExoS54-Cre	54 aa of ExoS fused to nuclear Cre recombinase in pUCP20; Cb^r^	This Study
pExoS96-Cre	96 aa of ExoS fused to nuclear Cre recombinase in pUCP20; Cb^r^	This Study
pExoS234-Cre	234 aa of ExoS fused to nuclear Cre recombinase in pUCP20; Cb^r^	This Study
pExoS-Flag	pHW0224, pUCP18 containing catalytically inactive ExoS with a Flag tag; Cb^r^	[34]
pExoS54	N-terminal 54 aa of ExoS in pUCP20; Cb^r^	This study
pEX18Tc	Vector containing *sacB* and Tc^r^ for exconjugant selection	[35]
pEX18Tc-ΔS	pEX18Tc containing 1 kb regions up and downstream of *exoS*; Tc^r^	This Study
pEX18Tc-ΔT	pEX18Tc containing 1 kb regions up and downstream of *exoT*; Tc^r^	This Study
pEX18Tc-ΔY	pEX18Tc containing 1 kb regions up and downstream of *exoY*; Tc^r^	This Study

The ExoS-Cre recombinase fusion constructs were generated by PCR amplification of successive lengths of the 5′ region of *exoS*, using primers listed in Table 2. Each primer contains 5′ restriction endonuclease sites (*Eco*RI or *Sac*I) for convenient cloning. The nuclear Cre recombinase gene was amplified to include an SV40 large T antigen nuclear localization sequence [26] using the primers listed in Table 2. Cre recombinase and a variable portion of *exoS* were cloned into pUCP20 in a triple ligation using *Eco*RI, *Sac*I and *Sal*I restriction sites. Constructs were confirmed by restriction analysis and DNA sequencing. *P. aeruginosa* PAK-JΔ*STY* was generated by successive unmarked allelic exchange, as previously described [27].

**Table 2:** PCR primers used in this study.

**For ExoS-Cre fusions**	
ExoS- upstream	5′-GACGAATTCGGCGTCTTCCGAGTCACTGGAGGC-3′
ExoS17 downstream	5′-GACGAGTCGTGCAATTCGACGGCGAAAGACGG-3′
ExoS54 downstream	5′-GAGCTCGAGCAGCCCCTCACCCTTCGGCGCGTCC-3′
ExoS96 downstream	5′-GACGAGCTCGGACATCAGCGCAGGCTGCGCGTC-3′
ExoS129 downstream	5′-GACGAGCTCTTCCGGTGTCAGGGTCGCCAGCTC-3′
ExoS234 downstream	5′-GACGAGCTCCTTGTCGGCCGATACTCTGCTGAC-3′
ExoS full downstream	5′-GACGAGCTCGGCCAGATCAAGGCCGCGCATCCT-3′
Cre upstream	5′-GGAGCTCATGCCTAAGAAGAAACGAAAGATC-3′
Cre downstream	5′-CGAGGTCGACGGTATCGATAAGCTTG-3′
**For ** ***exoS*** **, ** ***exoT*** ** and ** ***exoY*** ** deletion**	5′ and 3′ PCR primer sets
ExoS upstream(*Eco*R1-*Bgl*II)	5′-CAAGGAATTCGGATTATGCGGAGGGGTTGCCGGTG-3′ 5′-GTTGAGATCTCCTGATGTTTCTCCGCCAGTCTAGGAA-3′
ExoS downstream(*Bgl*II-*Hind*III)	5′-GTCCAGATCTTGGCTCGGCAGCGGATCCGGGTGGAG-3′ 5′-TGGAAAGCTTCGTCATCCTCAATCCGTACGGCAGGC-3′
ExoT upstream(*Eco*R1-*Bam*H1)	5′-GGAGGAATTCGAAGGGGTTGCGCAGGCCTGGCTCGTC-3′ 5′-TGACGGATCCTGATGTTTCCCCGCCAGTCTAGGAACG-3′
ExoT downstream(*Bam*H1-*Hind*III)	5′-CGGAGGATCCCAAGGGGTGTCCGTTTTCATTTGCGCC-3′ 5′-AGGTAAGCTTCCAGCGCCTGCGCCTGGGCCTCCTTG-3′
ExoY upstream(*Eco*R1-*Bam*H1)	5′-AACTGAATTCCGAGGATGTCGCCCTGCTCGACCATCGGG-3′ 5′-CCCAGGATCCAGGAGGCGCTCGACTTTTTCCAACGTA-3′
ExoY downstream(*Bam*H1-*Hind*III)	5′-ATAAGGATCCGGGCAGCGGCGAGATATCAGAAAACG-3′ 5′-CGTTAAGCTTGAGATAGCCGAGCATGCTCAGGCCGTC-3′

### Cell Culture

TE26 cells, a single cell clone carrying floxed nuclear *lacZ* reporter gene derived from human rhabdomyossarcoma cell line TE671 (ATCC CRL08805), were grown in Dulbecco's Modified Eagle Medium (DMEM; Gibco) supplemented with 15% Fetal Bovine Serum (FBS) [28]. R26R-EYFP mouse embryonic stem cells (mESC) [27] and TNGim05 induced pluripotent stem cells (iPSC) [17], were grown on 0.1% gelatin (Millipore) coated plates in mESC media [29]. All cells were cultured at 37°C with 5% CO_2_, and supplemented with Penicillin, Streptomycin, and Amphotericin (Cellgro). Gentamicin and Ciprofloxacin were added at final concentrations of 200 µg and 20 µg per ml, respectively, where noted.

### Protein injection assay

Mammalian cells were plated at approximately 60% confluency in antibiotic-free media the night before infection. *P. aeruginosa* was grown at 37°C in L broth containing carbenicillin until reaching OD_600_ of 0.8. Cells were infected with 0.5×10^8^ cfu *P. aeruginosa* per ml of growth media for a multiplicity of infection (MOI) of 50. Cells were co-incubated with *P. aeruginosa* for 3 hours in DMEM containing 5% FBS, unless otherwise stated. Infections were cleared by removing the media and washing cells 3 times in PBS, and adding media containing gentamicin and ciprofloxacin.

For Western Blot analysis of translocated proteins, cells were collected immediately following infection by incubation with 0.25% Trypsin for 5 minutes. The suspension was centrifuged for 5 minutes at 500 xg. Cells were washed in PBS, and centrifuged for 5 minutes at 500 xg. The cell pellets were lysed in 40 µl PBS containing 0.25% Triton-X on ice for 10 minutes. The lysed cells were then centrifuged at 16,000 xg for 5 minutes. The soluble fraction was collected, mixed with an equal volume of 2x SDS-PAGE loading buffer and boiled for 10 minutes. Following separation on 12% SDS-PAGE and transferred to PVDF, The blots were probed with antibodies against ExoS (rabbit polyclonal), Cre recombinase (mouse monoclonal, Abcam), or Flag (mouse M2 monoclonal Ab, Sigma).

### Immunocytochemistry

Cells were fixed in 3.7% formaldehyde in PBS for 15 minutes at room temperature. Cells were then washed 3x in PBS and permeablized in 0.5% Triton X-100 in PBS. Cells were then washed 3x in PBST and blocked with 1% BSA in PBST for 30 minutes. Cells were incubated with primary antibodies for 2 hours at room temperature, then washed 3x in PBST. Cells were incubated with the secondary antibody for 1 hour at room temperature, then washed 3x in PBST and examined under fluorescence microscope.

### Cytotoxicity assays

Cells were infected by *P. aeruginosa* for 1, 2, or 3 hours as described above. After infection, the cells were washed and incubated with 0.25% Trypsin for 5 minutes. The number of cells were then counted under microscope. The LDH release assay used CytoTox96 (Promega) and followed the manufacturer's instruction.

### β-galactosidase assays

TE26 cells were stained for β-galactosidase activity 48 hours after *P. aeruginosa* infection. Cells were fixed in 1% formaldehyde and 0.2% gluteraldehyde in PBS for 5 minutes and stained in a solution containing 4 mM K_4_Fe(CN)_6_, 4 mM K_3_Fe(CN)_6_, 2 mM MgCl_2_ and 0.4 mg/ml X-gal for 24 hours at 37°C. Percent β-galactosidase positive cells were determined by visual counts of blue and white cells under a light microscope.

### Flow cytometry

Infected cells were collected by 0.25% Trypsin treatment 48 hours after *P. aeruginosa* infection. Cells were centrifuged at 500 xg for 5 minutes and resuspended in 0.5 ml PBS containing 2% FBS. Cells were analyzed for EYFP or mOrange expression using Diva v6.2 on LSR-II (BD-Biosciences) and FACS Aria II (BD-Biosciences) for flow cytometry.

### Cell synchronization

TE26 cells grown in DMEM containing 15% FBS were cultured for 2 days and then incubated in medium containing 2 mM thymidine for 18 hours. Cells were then washed twice with PBS, and incubated with regular DMEM for 9 hours before a second incubation in 2 mM thymidine for 17 hours. At 0, 2, 5, 6, 9, 12, 16 and 24 hours after release from the second thymidine block, cell-cycle phase distribution was analyzed by flow cytometry with propidium iodide (PI) staining to verify the synchrony. Briefly, cells were fixed with ice-cold 70% ethanol for 24 h, then centrifuged and the cell pellet resuspended in 0.4 ml of PBS, 50 µl of RNase A (10 mg/ml) and 10 µl of PI (2 mg/ml). The mixture was incubated in the dark at 37°C for 30 min and then analyzed by FACsort flow cytometer (Becton Dickinson).
